# Focusing on Phosphorus Loads: From Healthy People to Chronic Kidney Disease

**DOI:** 10.3390/nu15051236

**Published:** 2023-02-28

**Authors:** Mengjing Wang, Jiaying Zhang, Kamyar Kalantar-Zadeh, Jing Chen

**Affiliations:** 1 Nephrology, Huashan Hospital, Fudan University, Shanghai 200040, China; 2 National Clinical Research Center for Aging and Medicine, Huashan Hospital, Fudan University, Shanghai 200040, China; 3 Nutritional Department, Huashan Hospital, Fudan University, Shanghai 200040, China; 4 Harold Simmons Center for Kidney Disease Research and Epidemiology, Division of Nephrology and Hypertension, University of California Irvine Medical Center, Orange, CA 92868, USA; 5 Fielding School of Public Health at UCLA, Los Angeles, CA 90095, USA; 6 Los Angeles Biomedical Research Institute at Harbor-UCLA, Torrance, CA 90502, USA

**Keywords:** phosphorus load, chronic kidney disease, phosphorus pool

## Abstract

Phosphorus is an essential micromineral with a key role in cellular metabolism and tissue structure. Serum phosphorus is maintained in a homeostatic range by the intestines, bones, and kidneys. This process is coordinated by the endocrine system through the highly integrated actions of several hormones, including FGF23, PTH, Klotho, and 1,25D. The excretion kinetics of the kidney after diet phosphorus load or the serum phosphorus kinetics during hemodialysis support that there is a “pool” for temporary phosphorus storage, leading to the maintenance of stable serum phosphorus levels. Phosphorus overload refers to a state where the phosphorus load is higher than is physiologically necessary. It can be caused by a persistently high-phosphorus diet, renal function decline, bone disease, insufficient dialysis, and inappropriate medications, and includes but is not limited to hyperphosphatemia. Serum phosphorus is still the most commonly used indicator of phosphorus overload. Trending phosphorus levels to see if they are chronically elevated is recommended instead of a single test when judging phosphorus overload. Future studies are needed to validate the prognostic role of a new marker or markers of phosphorus overload.

## 1. The Distribution of Phosphorus

Phosphorus is the sixth most abundant element in the human body and plays a key role in cellular metabolism and tissue structure [1]. Most body phosphorus (85%) is stored in the bones and teeth. The remainder (14%) is stored in soft tissues. Only 1% of the body’s total phosphorus stores are found in the extracellular fluid (ECF, including serum) [2]. In skeletal tissue, phosphorus is primarily complexed with calcium in the form of hydroxyapatite crystals, while the remaining phosphorus is found in the form of amorphous calcium phosphate [3]. In soft tissue, phosphorus exists mainly as phosphate esters (and, to a lesser extent, as phosphoproteins and free phosphate ions) [4]. Phosphorous is involved in a wide variety of essential cellular functions. These include biochemical energy transfer via adenosine triphosphate (ATP), maintenance of genetic information within DNA and RNA nucleotides, intracellular signaling via cyclic adenosine monophosphate (cAMP), and membrane structural integrity via glycerophospholipids. The ECF is responsible for the transport of phosphorus to and from the organs involved in phosphorus metabolism [5]. As a part of ECF phosphorus, serum phosphorus is easily measurable and can signal the status of body phosphorus stores. The normal range for serum phosphorus in adults is 2.5–4.5 mg/dL [0.81–1.45 mmol/L], but varies with age (the younger the child, the higher the normal phosphorus level) [3].

## 2. Phosphorus Homeostasis and In-Depth Review of Phosphorus Regulation

**Figure 1:**
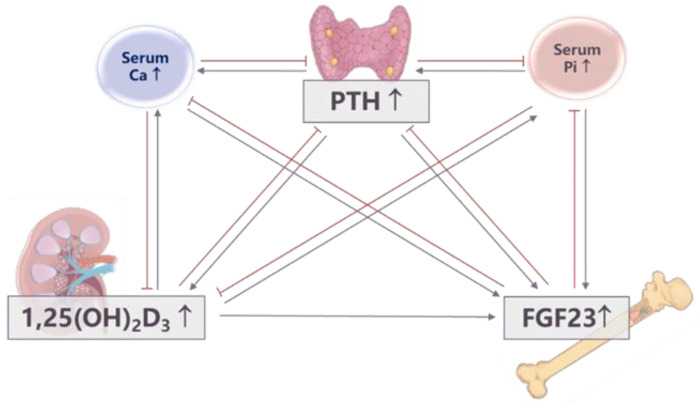
Phosphorus regulatory network. Note: Fibroblast growth factor 23 (FGF23), parathyroid hormone (PTH), and 1,25(OH)2D3 responds to the phosphorus load by modulating intestinal phosphorus absorption, urinary phosphate excretion, and phosphate distribution into bone. In addition, these hormones are capable of modulating one another’s secretion in vitro and in vivo animal models, thus forming a complex phosphorus regulatory network.

**Figure 2:**
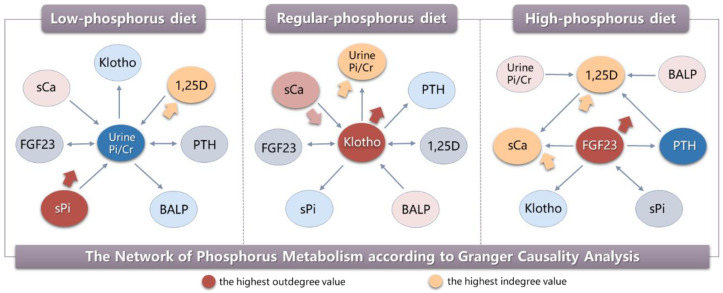
The network of phosphorus metabolism using Granger causality analysis following the low-, regular-, and high-phosphorus diet interventions. Note: Causality graph with the highest betweenness and degree effects following three diet interventions. Betweenness centrality is often used to find nodes that serve as a bridge from one part of a graph to another. The parameter with the highest betweenness was in the center of each graph with all the causal connections associated with this parameter. The thin arrows show the regulatory relationships associated with this phosphatonin. The big orange and red arrows indicate parameters with the highest outdegree and indegree values. Parameters with the highest outdegree value have the most regulating effect over other parameters in phosphorus metabolism. Parameters with the highest indegree value means they were mostly regulated. The head of the arrow represents the cause, and the tail represents the result. Abbreviations: Urine Pi/Cr, urinary phosphorus/creatinine rate; PTH, parathyroid hormone; FGF23, fibroblast growth factor 23; BALP, bone alkaline phosphatase; 1,25D, 1,25-dihydroxyvitamin D3; sPi, serum phosphorus; sCa, serum calcium.

Because phosphorus is involved in a series of physiological activities, maintaining its homeostasis is very important. The intestines, bones, and kidneys are the primary organs that maintain phosphorus homeostasis and control phosphorus concentration in circulation [6,7]. They perform this function via three endocrine system hormones: fibroblast growth factor 23 (FGF23), the parathyroid hormone (PTH), and 1,25(OH)_2_D_3_ (1,25D) [8,9,10,11,12,13,14]. It is well known that these three hormones respond to phosphorus loads by modulating intestinal phosphorus absorption, urinary phosphorus excretion, and phosphorus distribution into bone. The most typical is oral phosphorus administration, which caused significant increases in FGF23 by 2 h and PTH by 4 h in non-DM patients [15]. These hormones are also capable of modulating each other’s secretion in in vitro and in vivo animal models, thus forming a complex phosphorus regulatory network [16,17,18,19] (Figure 1). It has also been reported that Klotho has both FGF23-independent and FGF23-dependent roles in phosphorus homeostasis [20]. However, it is unknown how this reciprocal regulation network works under different physiological states. Granger causality analysis is a statistical method for investigating the flow of information between time series [21]. It is superior for resolving the construction and interaction of complex networks and for revealing causality relationships among multiple variables. The Granger causality methodology was first developed in econometrics, but has been widely applied to many other fields, including cardiology and neuroscience [22,23]. We recently used Granger causality analysis in a prospective clinical study that was attempting to determine the most important initiating factors, mediating factors, and outcomes involved in in vivo phosphorus regulation. Six healthy men were enrolled and treated with different phosphorus diets for five days, with urine collection and blood drawn every two hours on the last day of the diet for biochemical analysis. Granger causality analysis showed that serum αKlotho was the most strongly connected variable, and that it played a key role in influencing other factors. In addition, urinary phosphorus excretion was frequently regulated by other factors in the phosphorus regulatory network after regular-phosphorus (1500 mg/d) diets. After low-phosphorus (500 mg/d) diets, serum phosphorus affected other factors, with 1,25D being the main output factor, while urinary phosphorus excretion was the most strongly connected variable in the phosphorus metabolic network. After high-phosphorus (2200 mg/d) diets, both serum FGF23 and 1,25D played critical roles in active and passive phosphorus regulation (Figure 2) [24]. These results illustrated how the dominant mechanisms of phosphorus regulation differ under different dietary conditions and suggest that the precise phosphorus regulatory network mechanisms need to be further explored.

**Figure 3:**
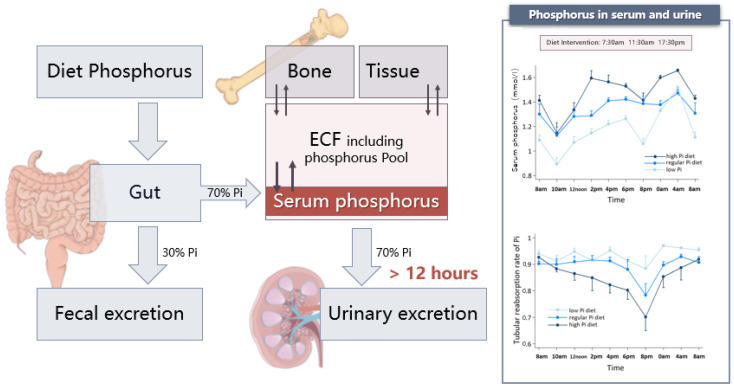
Phosphorus transport among three organs, tissue, and ECF. Note: Gut and kidney handle the major fraction of daily phosphorus transport for absorption and excretion. Urinary excretion of phosphorus lasts for at least 12 h to finally reach phosphorus balance. The phosphorus pool acts as a buffer pool throughout the slow excretion process of the kidney to ensure that the serum phosphorus is in a narrow range. The arrows mean the exchange between different tissues or organs.

Amongst the three major organs, the gut and kidney handle the major fraction of daily phosphorus transport for absorption (70% of dietary phosphorus) and excretion (70% of dietary phosphorus), respectively [5,25,26] (Figure 3). However, when there are large gaps between the total amount of phosphorus that needs to be excreted and the rate of phosphorus excretion, it can take the kidney a long time to excrete the absorbed dietary phosphorus. For example, if a regular diet contains 1500 mg of phosphorus, the kidney will need 20 h to excrete the absorbed 980 mg of phosphorus at a rate of 45 mg per hour based on a tubule reabsorption rate of over 80% [27]. When the tubule reabsorption rate decreases as a response to PTH or FGF23, the excretion may accelerate. We also observed this extended excretion phenomenon in the aforementioned cross-over clinical trial. Under regular- and high-phosphorus diet conditions, the phosphorus tubule reabsorption rate decreased but still lasted over 12 h (Figure 3). Following a high-phosphorus diet, however, there was a longer duration of tubule reabsorption rate changes, indicating a 24-h excretion (Figure 3). Several other studies have shown similar findings. Nishida observed that the cumulative urinary excretion of phosphorus continues to increase even 8 h after a single oral phosphorus load [28]. This is also true in the case of intravenous phosphorus loading. Scanni found that the urinary excretion of phosphorus lasted for at least 12 h and that fractional phosphorus clearance kept increasing for 12 h after a 36-h parenteral or duodenal acute phosphorus load, even though serum phosphorus significantly decreased at the 36th hour [29]. Thus, we infer that absorbed phosphorus from diets are likely first stored somewhere in the body so that the kidney can gradually excrete them. This is another important and perhaps overlooked aspect of phosphorus homeostasis. However, this slow excretion could not be explained by slow digestion and/or absorption because we know that intestinal absorption is completed approximately 7.5 h after intake [26]. It may be associated with the circadian rhythm of serum phosphorus, cortisol, or PTH regulation, which could explain the peak and trough concentrations of serum phosphorus and the obvious drop in tubular phosphorus reabsorption that we observed at 8:00 pm no matter what diet the patients were on (Figure 3) [30,31,32,33,34,35]. Thus, it is clear that phosphorus regulation and homeostasis are much more complicated than a network composed of phosphatonins. An after-diet phosphorus storage, with a corresponding as yet unidentified mechanism, must also be involved.

## 3. Phosphorus Pool

Long bone hydroxyapatite crystal serves as a huge phosphorus reservoir that can be rapidly mobilized to support numerous biological systems. Daily transport of phosphorus (180 mg) from the ECF to form new bones formation and then back to ECF via bone resorption keeps phosphorus balanced, but involves a far smaller percentage of total body phosphorus than the amount that is regulated by the gut and kidney [5,25,26]. Thus, the latter are the main organs that regulate phosphorus homeostasis. The delays in renal excretion of phosphorus after dietary intake suggest that there might be a “pool” for temporary phosphorus storage, leading to the continuous release of phosphorus from the pool to the kidney and the maintenance of stable serum phosphorus levels. The “phosphorus pool” theory was firstly proposed by Spalding et al. when studying phosphorus kinetics during hemodialysis [36,37]. Spalding et al. explained that a third phosphorus pool usually switched on to release phosphorus into the extracellular space to protect the intracellular phosphorus concentrations from decreasing too low because intracellular phosphorus plays critical roles in energy-dependent processes, intracellular buffering, and protein activity regulation [36]. Agar et al. also demonstrated evidence of undefined phosphorus pools from which phosphorus was continuously mobilized into the ECF at a dynamic mobilization clearance rate during dialysis [38]. More recently, after adaption by Daugirdas et al., the above model/theory could be used to predict intra-dialysis and early post-dialysis serum phosphorus values, adding further evidence to the idea that there is a phosphorus storage pool [39].

**Figure 4:**
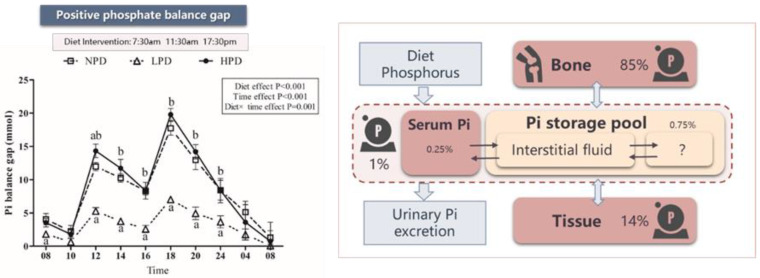
The storage pool severs as a buffer pool for the transient positive phosphorus balance gap. Note: The phosphorus balance gap in the left half of this figure was calculated as follows: phosphorus balance gap (mg) = phosphorus balance gap at the previous time point (mg)—the urinary phosphorus excretion at the current time point (mg). Phosphorus balance gap at 08:00 was the first gap. It was calculated as the dietary phosphorus intake of breakfast multiplied by the intestinal absorption rate—the urinary phosphorus excretion at 08:00 (mg). As for phosphorus balance gap at 12:00 and 18:00, the corresponding dietary phosphorus intake per meal multiplied by the intestinal absorption rate were added to the above first formula. Intestinal absorption rate was calculated by 24-h urinary phosphorus excretion divided by dietary phosphorus intake. The arrows in the right half of the figure mean the exchange between different tissues or organs. Abbreviations: LPD, low-phosphorus diet (500 mg/d), NPD, normal-phosphorus diet (1500 mg/d), HPD, high-phosphorus diet (2200 mg/d).

Our cross-over clinical trial in healthy adults also supports these findings. When we calculated phosphorus balance gaps (i.e., the absorbed dietary phosphorus minus the amount of urinary phosphorus excretion), we found that positive phosphorus balance gaps last for almost the whole day—from 08:00 to 08:00 the next day [40] (Figure 4). Although there was a small increase in serum phosphorus levels, the extra phosphorus stored in the ECF was actually much smaller than the phosphorus gap. Most of the phosphorus absorbed from the intestine needed to be stored in preparation for excretion (Figure 4). Thus, we suspect that, in physiological states, the storage pool serves as a buffer to avoid too rapid phosphorus increases and to maintain stable intracellular phosphorus ranges.

To date, the source of the storage pool (“third pool”) still remains unknown. We believe that this pool is part of the ECF, and thus minimally affects organ and/or tissue metabolism. One previous research group hypothesized that this phosphorus was not yet incorporated into the bone [36]. A recent study on changes in phosphorus distribution during hemodialysis raised the possibility of intracellular phosphorus storage in muscles since muscles have high intracellular phosphorus concentrations and could tolerate between 30–40% reductions in phosphorus concentrations without exhibiting bioenergetics [41]. However, Bevington et al. found that the percentage change in intramuscular phosphorus only increased by 70%, even when the plasma phosphorus concentration range changed fourfold [42]. Additionally, this theory still cannot explain the persistent rebound of serum phosphorus after dialysis because dialysis also decreases intracellular muscle tissue Pi concentrations by up to 23%, a percentage that has been described in the muscle tissue of patients with chronic obstructive pulmonary disease [43,44].

## 4. Phosphorus Overload and Pathological Phosphorus Pool

Phosphorus loading refers to the amount of phosphorus intake into a system [1]. For example, dietary phosphorus is one of the main kinds of phosphorus loading. Dietary phosphorus could cause a transient increase in the phosphorus storage pool, but it is eventually excreted by the kidney under the control of the phosphorus regulatory network. In cases where phosphorus loadings exceed the kidneys’ excretion threshold, the physiological phosphorus pool may become saturated. This excess phosphorus can lead to vascular calcification or hyperphosphatemia [45]. We define hyperphosphatemia as a type of phosphorus overload which leads to the formation of pathological phosphorus pools in the vasculature and subsequent cardiovascular disease. A typical example of this situation is CKD. As renal phosphorus excretion decreases, serum phosphorus and coronary artery calcification scores gradually increase [46,47]. Therefore, a precise assessment of the phosphorus overload is very important. However, hyperphosphatemia is not a sensitive indicator of phosphorus overload. Clinical studies on dietary phosphorus have found that, after a high-phosphorus diet, most people still have normal ranges of serum phosphorus. We thus suggest the following formula to help recognize true phosphorus overload: phosphorus absorbed from the intestine—the phosphorus excretion from urine + net phosphorus absorption/release from osteogenesis and osteolysis + net phosphorus absorption/release from tissue metabolism (Figure 4). Phosphorus overload may be difficult to define on the basis of a specific value since the demand for phosphorus changes as physiologic states change. However, we can prove phosphorus overload exists on the basis of pathological changes (i.e., hyperphosphatemia and vascular calcification).

## 5. Reasons for and Deleterious Side Effects of Phosphorus Overload

Exploring the underlying causes of phosphorus overload is important to reduce unwanted side effects. One potential cause is longstanding abnormalities in any element of the phosphorus regulatory network. Dietary phosphorus may be the main source of phosphorus overload in young adults with normal kidney function and bone turnover. Renal function decline and osteoporosis may be the main causes of phosphorus overload in the elderly because the glomerular filtration rate (GFR) tends to decrease as a part of the normal aging process and in cases of chronic disease [48,49]. Serum phosphorus could be decreased by denosumab, which induces reduction in phosphorus load into the bones of osteoporotic patients [50]. Additionally, the age-adjusted prevalence of low bone mass can be as high as 43.1% among adults aged 50 and over [51]. For CKD patients, an inappropriate diet, compromised renal function, renal osteodystrophy, insufficient dialysis, and prescription medications could all cause phosphorus overload. In particular, phosphorus overload happens early in the CKD disease course (i.e., Craver et al. found decreases in the 24-h urine phosphorus excretion of CKD patients), but intestinal phosphorus absorption appears to be near normal in CKD patients [46,47].

**Figure 5:**
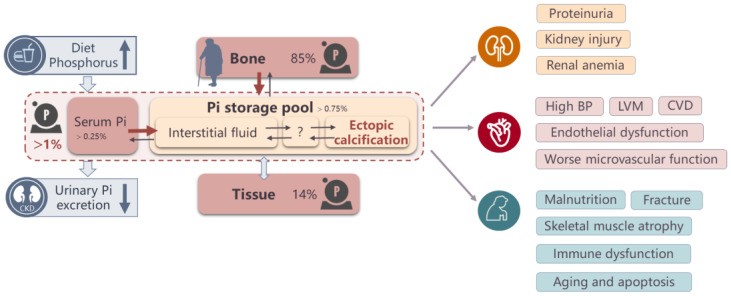
Reasons for phosphorus overload and harm of phosphorus overload. Note: Phosphorus balance can easily be disequilibrated by a persistent high-phosphorus diet, renal function decline, bone disease in the young population, CKD patients, and the elderly, and can finally result in phosphorus overload. Phosphorus overload leads to a serious of side effects including hyperphosphatemia, vascular calcification, a worse microvascular function, and so on. These are also the indirect evidence of phosphorus overload. The arrows mean the exchange between different tissues or organs.

Substantial evidence from epidemiological studies indicates that phosphorus overload is associated with poor outcomes (Figure 5). High dietary phosphorus is associated with higher blood pressure [40,52], greater left ventricular mass [53], more severe proteinuria [54,55,56,57], renal calcification [58], intraglomerular hypertension and proximal tubular injury [59,60], fractures [61], and higher mortality [62]. A low bone mineral content has been shown to be a risk factor for increased incidence of coronary artery disease and cardiovascular mortality [63,64]. Osteoporosis blocks the skeleton from exerting its reservoir function when positive phosphorus balance occurs, and is also associated with heterotopic mineralization [65,66,67,68]. As a direct indicator of phosphorus overload, higher serum phosphorus concentrations, even within the normal range, are independently associated with worse microvascular function, coronary artery calcification, incident CKD, and mortality in community-living individuals [69,70,71,72].

In CKD, the harm of phosphorus overload appears earlier and has more severe consequences than in healthy controls (Figure 5). Excess phosphorus exerts toxic effects through a variety of pathways, including direct effects of hyperphosphatemia and indirect effects related to compensatory responses, such as increased FGF23 and PTH levels. With the exception of calcification, high serum phosphorus levels directly potentiate endothelial dysfunction, promote the progression of kidney disease, and induce cellular stress, premature aging, and apoptosis [1,73]. High levels of FGF23 and PTH induce left ventricular hypertrophy, renal anemia, immune dysfunction, adipose tissue browning, and skeletal muscle atrophy [74]. Enhanced urinary phosphorus excretion, as a compensatory reaction for high dietary phosphorus loads, also leads to progressive nephron loss via calcium phosphorus particle-induced damage to tubule cells [59,75]. Thus, phosphorus overload should be assessed and treated as quickly as possible, even when normophosphataemia is suggested based on FGF23 or PTH levels.

## 6. Early Markers of Phosphorus Overload

**Figure 6:**
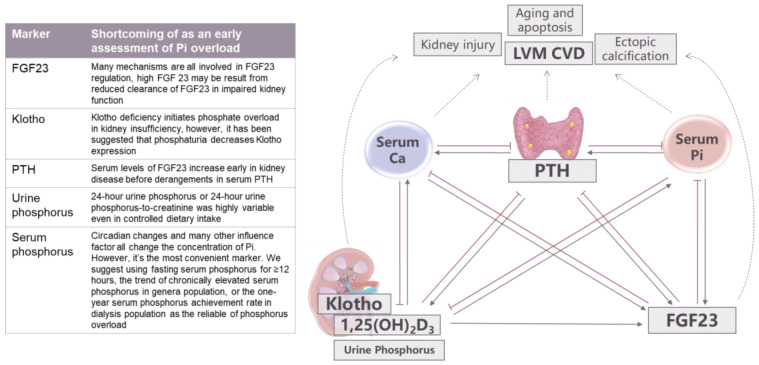
Early markers of phosphorus overload. Note: The arrows represent the promotion or inhibition effects.

The early detection of phosphorus overload improves long-term outcomes. However, what regulatory processes first respond to high phosphorus loads and how the extent of the response is determined continue to be points of discussion. For example, in CKD, the classic hypothesis suggested that there was a trade-off between PTH for phosphorus level normalization and that this process occurred before hyperphosphatemia [76]. However, the discovery of the FGF23/Klotho system has improved our understanding of phosphorus disorders. A high phosphorus diet and high extracellular phosphorus levels increase serum levels of FGF23 early in kidney disease before there are changes in serum phosphorus and PTH [77,78,79]. However, FGF23 is probably not a reliable biomarker for phosphorus loads because PTH, calcitriol, calcium [80,81,82], erythropoietin (EPO), hypoxia-inducible factors (HIFs), various inflammatory stimuli, and kidney clearance are also involved in FGF23 regulation [83,84]. Early in the sequelae of kidney injury, Klotho expression is downregulated [85]. Klotho deficiency upregulates Na+-dependent phosphorus transporter 2a (NaPi-2a) and NaPi-2c expressions in the kidney and upregulates NaPi-2b in the intestines, all of which may initiate phosphorus loading in kidney insufficiency [20]. Interestingly, it has been suggested that phosphaturia decreases Klotho expression through the activation of the Wnt/β-catenin pathway [86]. Thus, the urine phosphorus creatinine ratio or a 24-hour phosphorus measurement may be better choices for predicting phosphorus loads. However, 24-h urine phosphorus measuring, or 24-h urine phosphorus-to-creatinine ratios, are highly variable in CKD patients, even those with tightly controlled dietary intakes [87] (Figure 6). Using daily intake minus fecal and urinary outputs to calculate phosphorus retention is an accurate method if bone and tissue are in metabolic balance. However, this procedure is too complicated to be widely applied in clinical practice. Additionally, 50–80% of the CKD population have comorbid bone disorders, meaning this approach could not be applied to most CKD patients [88,89,90]. Thus, optimizing current indicators or testing multiple potential indicators of phosphorus overload may be a good future direction. Our understanding of the role of serum phosphorus could be improved. Assessment of phosphorus overload based on a single serum phosphorus level should be avoided since there are not only circadian changes in serum phosphorus levels but also changes based on diet, dialysis, and drugs [74]. This also partially explains the different nadirs of serum phosphorus found in relation to mortality [91,92]. Fasting serum phosphorus for ≥12 h excludes the “noise” of dietary intake and may be a better choice, as it has been demonstrated to be associated with increased mortality greater than measuring <12 h serum phosphorus levels [93]. Chronically elevated serum phosphorus levels, even in the normal range, are likely an expression of a prolonged exposure to phosphorus overload. If serum phosphorus was regularly followed in hemodialysis patients, the one-year serum phosphorus achievement rate (defined as the number of tests within the target range [2.5–4.5 mg/dL] divided by the total number of tests given throughout a year) may be a better indicator than one-time phosphorus or averaged phosphorus levels [94]. Keeping the one-year achievement rate of serum phosphorus higher than 50% provides significant clinical benefits in reducing cardiovascular mortality [94].

## 7. The Path Forward

Evidence related to early control phosphorus overload is limited since we have historically focused on treating hyperphosphatemia. Controlling potential phosphorus overloads needs to be a high priority, especially among patients who are not prone to hyperphosphatemia. A few interventions, including dietary phosphorus restriction and phosphorus-binder therapy, have been tested in CKD stage 3/4 patients with normophosphatemia. Studies on diet control showed that low phosphorus diets were associated with higher serum 1,25D levels and lower serum Pi and FGF23 values, which slowed the progression of CKD [95,96,97]. RCTs on phosphorus load management with phosphorus binders in CKD patients have yielded conflicting results in terms of the long-term benefits, however [98,99,100]. These indicate that the use of unreliable markers of phosphorus overload may preclude any definite conclusions [101]. Therefore, by analyzing the regulation of phosphorus metabolism networks and clarifying the compensatory mechanism and underlying causes of phosphorus overload under different conditions, we can control phosphorus overload in a targeted manner. We have also demonstrated that, in hemodialysis patients, the bone disease still contributes the most to phosphorus burdens, followed by dietary factors [94], suggesting that actions may be taken on the basis of bone disease markers. However, in hemodialysis (HD) patients with substantial residual kidney function (RKF), RKF was the only independent predictor of serum phosphorus levels [102]. This emphasizes the importance of protecting RKF in incident hemodialysis patients, which can be performed in several ways, such as starting with incremental dialysis [103,104,105,106]. Finally, we speculate that every person may have his/her own ideal level of serum phosphorus, but whether the percentage of increase from the basal levels can guide phosphorus overload control and improve prognoses needs further validation.

## 8. Conclusions

Phosphorus metabolism involves at least three main organs: the intestine, kidneys, and bone. The endocrine regulation of Pi metabolism is completed via the highly integrated actions of several hormones, including FGF 23, PTH, Klotho, and 1,25D. Our previous study showed that a key regulatory factor of serum phosphorus varied under different phosphorus diets. Our study of dietary phosphorus interventions demonstrated that the kidney needs a long time to excrete dietary phosphorus. Combined with findings on phosphorus kinetics during hemodialysis, our results support the notion that a physiologic phosphorus pool exists and acts as a buffer for the storage of excess body phosphorus. The pool allows for the release of phosphorus into extracellular space and the maintaining of a stable serum phosphorus. The physiological phosphorus pool may not be associated with ill effects, since final phosphorus balances will be reached via kidney excretion under the control of the phosphorus regulatory network. However, phosphorus overload can lead to serious side effects, including the formation of pathological phosphorus pools. Potential causes of phosphorus overload include high-phosphorus diets, renal function decline, osteoporosis, insufficient dialysis, and prescription medications. Serum phosphorus remains the most commonly used indicator of phosphorus overload. We recommend against the use of a single phosphorus measurement, but chronically elevated serum phosphorus trends and/or the rate of in-target measurements over time can be used as markers of phosphorus overload in HD patients. However, by clarifying the details of the metabolic phosphorus regulatory network and the reasons for the phosphorus overload under different conditions, we can control phosphorus overload in a targeted manner. Measuring and targeting serum phosphorus levels on an individual basis may also be an important future direction.

## Data Availability

Data could be obtained via an email to the corresponding author under reasonable request.
